# Development of *de-novo* transcriptome assembly and SSRs in allohexaploid Brassica with functional annotations and identification of heat-shock proteins for thermotolerance

**DOI:** 10.3389/fgene.2022.958217

**Published:** 2022-09-16

**Authors:** Kaushal Pratap Singh, Preetesh Kumari, Devendra Kumar Yadava

**Affiliations:** ^1^ ICAR-Directorate of Rapeseed Mustard Research, Bharatpur, India; ^2^ Genetics Division, ICAR—Indian Agricultural Research Institute, New Delhi, India

**Keywords:** allohexaploid Brassica, illumina Novaseq 6000, RNA-seq, *de-novo* assembly, functional annotations, SSRs, gene ontology

## Abstract

Crop Brassicas contain monogenomic and digenomic species, with no evidence of a trigenomic Brassica in nature. Through somatic fusion (*Sinapis alba* + *B. juncea*), a novel allohexaploid trigenomic Brassica (H1 = AABBSS; 2*n* = 60) was produced and used for transcriptome analysis to uncover genes for thermotolerance, annotations, and microsatellite markers for future molecular breeding. Illumina Novaseq 6000 generated a total of 76,055,546 paired-end raw reads, which were used for *de-novo* assembly, resulting in the development of 486,066 transcripts. A total of 133,167 coding sequences (CDSs) were predicted from transcripts with a mean length of 507.12 bp and 46.15% GC content. The BLASTX search of CDSs against public protein databases showed a maximum of 126,131 (94.72%) and a minimum of 29,810 (22.39%) positive hits. Furthermore, 953,773 gene ontology (GO) terms were found in 77,613 (58.28%) CDSs, which were divided into biological processes (49.06%), cellular components (31.67%), and molecular functions (19.27%). CDSs were assigned to 144 pathways by a pathway study using the KEGG database and 1,551 pathways by a similar analysis using the Reactome database. Further investigation led to the discovery of genes encoding over 2,000 heat shock proteins (HSPs). The discovery of a large number of HSPs in allohexaploid Brassica validated our earlier findings for heat tolerance at seed maturity. A total of 15,736 SSRs have been found in 13,595 CDSs, with an average of one SSR per 4.29 kb length and an SSR frequency of 11.82%. The first transcriptome assembly of a meiotically stable allohexaploid Brassica has been given in this article, along with functional annotations and the presence of SSRs, which could aid future genetic and genomic studies.

## Introduction

Unlike naturally developed allohexaploid bread wheat (Gaebelein and Mason, 2018), trigenomic “allohexaploid Brassicas” with three genomes do not exist in nature (Mason and Batley, 2015). Such allohexaploid Brassicas have been successfully developed through somatic hybridization, and their potential is being examined, which is adapted to climatic change (Gupta et al., 2016; Wei et al., 2016). Higher ploidy levels are linked to speciation, gene augmentation, and genetic diversity (Leitch and Leitch, 2008; Soltis et al., 2009; Jiao et al., 2011; Tank et al., 2015) and provide genetic resources that can adapt to changing climatic conditions (Marchant et al., 2016). Although attempts to synthesize allohexaploid Brassica through interspecific hybridization have been made (Pradhan et al., 2010; Tian et al., 2010; Chen et al., 2011), such attempts have failed (Mason et al., 2012, 2014). However, wild diploid Brassicaceae members should be added to cultivated species as a source of unique genes for resistance/tolerance to a variety of fungal diseases, insects, pests, nematodes, heat, and drought (Sjodin and Glimelius, 1989; Kirti et al., 1995; Li et al., 2009; Garg et al., 2010; Singh et al., 2021). With this in mind, we developed stable allohexaploid Brassicas through somatic hybridization involving diploid *S. alba* (a Brassica coenospecies) and tetraploid *B. juncea* (an amphidiploid crop species); the resulting stable allohexaploids had a somatic chromosome number of 2*n* = 60 (AABBSS) and a high level of male and female fertility (Kumari et al., 2018; 2020c). Two allohexaploids (H1 and H2) showed resistance to Alternaria blight and Sclerotinia stem rot and had high-temperature tolerance (upto 40°C). The H1 allohexaploid also had recombinant mitochondria and *B. juncea*-type chloroplasts, which improved resistance to Alternaria blight and Sclerotinia stem rot (Kumari et al., 2018; Kumari and Singh, 2019), as well as several other desirable traits like thermotolerance, which is partly dependent on ubiquitous heat shock proteins (HSPs) and allows normal cell functions to be maintained during hot spells (Park and Seo, 2015). Under stressful conditions, they can aid to stabilize and refold proteins (molecular chaperons) (Whitley et al., 1999; Sitia and Braakman, 2003; Huttner and Strasser, 2012). HSPs are divided into five primary groups based on their size (kDa): HSP100, HSP90, HSP70, HSP60, and small HSP (sHSP) (Wang et al., 2004; Kotak et al., 2007; Gupta et al., 2010). To respond to stresses, HSPs can be found in the cytosol, endoplasmic reticulum, chloroplast, mitochondria, and nucleus (Vierling, 1991; Boston et al., 1996). The transcriptome profile studies in a large number of crop plants have been conducted to report these heat shock proteins such as Arabidopsis (Rahmati Ishka et al., 2018), rice (Sarkar et al., 2014), maize (Shi et al., 2017), and rapeseed (Wang et al., 2018a).

NGS technologies such as Illumina, Ion Torrent, PcaBio, Nanopore, and others are now being used for RNA sequencing (RNA-seq) for a number of objectives, including genomic architecture study, molecular pathway elucidation, and the production of molecular markers such as SSRs (Shawn and Richard, 2016; Zhang et al., 2017). However, no such research has been done on any stable allohexaploid Brassica (*B. juncea* + *S. alba*) yet. On the other hand, the allotetraploid Brassica napus was frequently used for transcriptome analysis to decipher the genic constitution for important agronomic and yield-related traits. Yao et al. (2020) used a single molecule long-read isoform sequencing (Iso-Seq) technique to unravel the complex nature of transcriptome in *B. napus*. Out of 147,698 unique long-read isoforms identified, a total of 37,403 genes were annotated. Moreover, An et al. (2019) used a different strategy to uncover the genic architecture of *B. napus* by using 183 *B. napus* accessions along with their diploid progenitors, i.e., *B. rapa* (112 accessions) and *B. oleracea* (62 accessions) and five other Brassicaceae members. These accessions collectively represented the complete phenotypic diversity of *B. napus* species and shared a total of 372,546 high-quality SNPs. The identification of a higher number of SNPs in the A subgenome than in the C confirmed the higher level of nuclear diversity. The transcriptome/RNA-seq strategy is successfully used to identify the target candidate gene for a particular trait. Jian et al. (2019) identified 115 flowering time–related differentially expressed genes that were related to plant circadian clock/photoperiod, autonomous pathway, and hormone and vernalization pathways. They identified a total of 27 quantitative loci dispersed on eight different chromosomes of *B. napus*. These loci were identified for harboring 45 candidate genes for flowering time.

In light of the foregoing, the current work was designed to examine the transcriptome of a stable allohexaploid Brassica that we had previously produced through somatic hybridization. The allohexaploid contains unique genes that confer disease resistance and heat stress tolerance. Transcriptome analysis can be used to characterize these new genes. We analyzed gene ontology (GO) and performed functional annotations against common protein databases. The transcriptome information is also used to create genic SSRs. The current research added to the knowledge of the genomic architecture of a synthetic allohexaploid Brassica. The findings of this study will serve as a genetic resource for future research on gene identification and expression patterns, population genetics, pathway investigations, phylogenetics, and marker-assisted selection (MAS).

## Materials and methods

### Plant material, total RNA isolation, and quality check

The allohexaploid (H1; Kumari and Bhat, 2021) was used as the experimental plant material in this investigation (Kumari et al., 2018). After the commencement of flowering in all field-grown plants, leaf samples were taken in triplicates from three individual plants in the evening for Total RNA isolation. Until RNA extraction, all samples were collected in liquid nitrogen and stored at −80°C. Total RNA was isolated from each sample using TRIzol Reagent (Invitrogen, CA, United States) according to the manufacturer’s instructions. Following Total RNA isolation, the samples were quality checked and quantified using the NanoDrop 8000 (OD260/OD280) and Qubit 3.0 Fluorometer (Invitrogen Life Technologies, United States), respectively, and qualified using a 1% agarose gel electrophoresis to check for RNA degradation and contamination.

### cDNA library construction, quality control, and RNA sequencing

All three RNA samples showed nanodrop ratio >2.0 and qubit concentrations 2170, 2240, and 2010 ng/μl, respectively. The equimolar concentration of Total RNA extracted from all three plant samples was combined to create the pooled sample and used for the downstream experiments. For the production of complementary DNA (cDNA) libraries (NEBNext II RNA Library Prep Kit for Illumina^®^), a total of 1 μg of Total RNA with a RIN (RNA integrity number) value greater than 7 was employed. Following the manufacturer’s instructions, the ready-to-run final library was quantified using a Qubit 3.0 fluorometer using a dsDNA HS assay kit (Q32851; Thermofisher, United States). The library’s insert size (391 bp) was determined using highly sensitive D1000 ScreenTapes5067-5582 on a TapeStation 4150 (Agilent Technologies, CA, United States) following the manufacturer’s protocol. Following the manufacturer’s directions, mRNA was purified from Total RNA using poly-T oligo-attached magnetic beads. The mRNA fragmentation buffer was added to break them into small fragments. Random primers were used to synthesize the cDNA strand from cleaved mRNA fragments during reverse transcription. DNA polymerase-I, dNTPs, buffer, and RNase H were used to make the second strand of cDNA. The freshly produced double-stranded cDNA was purified using the QIAquick PCR extraction kit (QIAGEN, Hilden, Germany) and washed with EB buffer. Adapters were ligated to the sequencing RNA on both ends and inserted adenine (A) nucleotide into the 3′ ends to repair the ends. AMPure XP beads were used to select fragments, which were then enriched by PCR to create a library for transcriptome sequencing. Nucleome Informatics Pvt. Ltd. in Hyderabad performed the RNA sequence library preparation and sequencing (India) (https://www.nucleomeinfo.com/). After pooling the qualified libraries according to their effective concentration and expected data volume, they were fed into an Illumina sequencer NovaSeq 6000 with S4 type Flow Cell (2 × 150 bp read length). After sequencing, the raw data in FASTQ format was used for *de-novo* assembly, microsatellite marker development, and functional annotations using bioinformatics tools. The raw data of the allohexaploid Brassica transcriptome sequence were submitted to the Sequence Read Archive (SRA) of the National Center for Biotechnology Information (NCBI) under accession number SRR14934389 and BioProject number PRJNA741791.

### *De-novo* assembly development, redundancy removal, and coding sequence extraction

*De-novo* assembly was constructed from clean reads with a quality value or Phred score (Q) of 30 after removing low-quality bases and adapters from 5′ and 3′ ends, very short sequences, and low-quality reads to yield robust transcripts of the allohexaploid Brassica. Bioinformatics tools like FastQC and NGS QC toolkits were used to clean the reads. The clean raw reads were assembled *de-novo* into transcripts using the Trinity v2.11.0 software (Grabherr et al., 2011), with default parameters, and k-mer 25, a short-read assembly program (https://github.com/trinityrnaseq/trinityrnaseq/wiki). After a successful run of the software, the assembly was utilized to filter out and identify true transcripts. The evidentiary gene packages (tr2aacds.pl) were used to remove the spurious transcripts while keeping the CDS that was at least 90 bp long (http://arthropods.eugenes.org/EvidentialGene/trassembly.html). The package was run following pipeline: Perfect redundant removal-fastanrdb (from the exonerate programme); perfect fragment identification- CD-HIT-EST –c 1.0 (https://github.com/weizhongli/cdhit); BLASTN to find highly similar transcripts; CDS classification- evigene/rnaseq/asmrna dupfilter2. Pl. The software-generated coding sequences (CDSs) were then used for microsatellite development and functional annotations. The completeness of the transcriptome assembly was also evaluated by OrthoDB database of orthologs to define BUSCO sets for eukaryote clade (https://www.orthodb.org).

### Functional annotations

OmicsBox v2.0 (https://www.biobam.com/omicsbox/) was used to mask the coding sequences for repeat sequence types, utilizing the RMBlast search engine and the Dfam v3.0 consensus repeat database, with the Brassicaceae family filter set to default values (Smit et al., 2013; Hubley et al., 2016). The masked CDSs were aligned with the combined protein database of *B. rapa* (GCF_000309985.2_CAAS_Brap_v3.01) and *Arabidopsis thaliana* (GCF_000001735.4_TAIR10.1) reference sequences downloaded from NCBI using local BLASTX at an expectation value of 1e-25 with default parameters. Through OmicsBox, the outcomes of the local blast search were used for downstream analyses, including mapping, annotations, gene ontology (GO), and pathway analysis (KEGG and Reactome). After BLASTX, the GO terms were classified into three categories: cellular component, molecular function, and biological process. The CDSs were also aligned using BLASTX (E 1e-25) against NCBI public databases, such as non-redundant protein sequences v5 (nr), reference proteins (refseq protein v5) using Brassicaceae, *B. juncea*, *B. rapa*, *B. nigra*, and *S. alba* as a taxonomic filter using OmicsBox and locally BLASTX against UniProtKB/Swiss-Prot v5 database on the CLC genomics workbench v20.0.4 (Qiagen, United States). Furthermore, utilizing the COG, KOG, Pfam (Stephen et al., 1990), Prk (Finn et al., 2010), and Tigrfam (Haft et al., 2003) protein databases (http://weizhong-lab.ucsd.edu/webMGA/), the protein translations of these CDSs were categorized and examined (Wu et al., 2011). To investigate the activities of putative genes against heat stress during seed development stages in allohexaploid Brassica, the CDSs were subjected to BLASTN with plant heat shock proteins (HSPs) retrieved from the NCBI Gene database. A total of 13,022 HSPs were downloaded, including A. thaliana, *H. syriacus*, *C. sativa*, *B. napus*, *B. rapa*, *H. annus*, *T. dicoccoides*, *P. sominferum*, and other plant genera included. The BLASTN tool was utilized through the CLC genomics workbench to identify the potential HSPs, with a 1e-25 expectation (E) value, a maximum number of hit sequences of 250, and word size of 11.

### SSR loci identification and marker development

**FIGURE 1:**
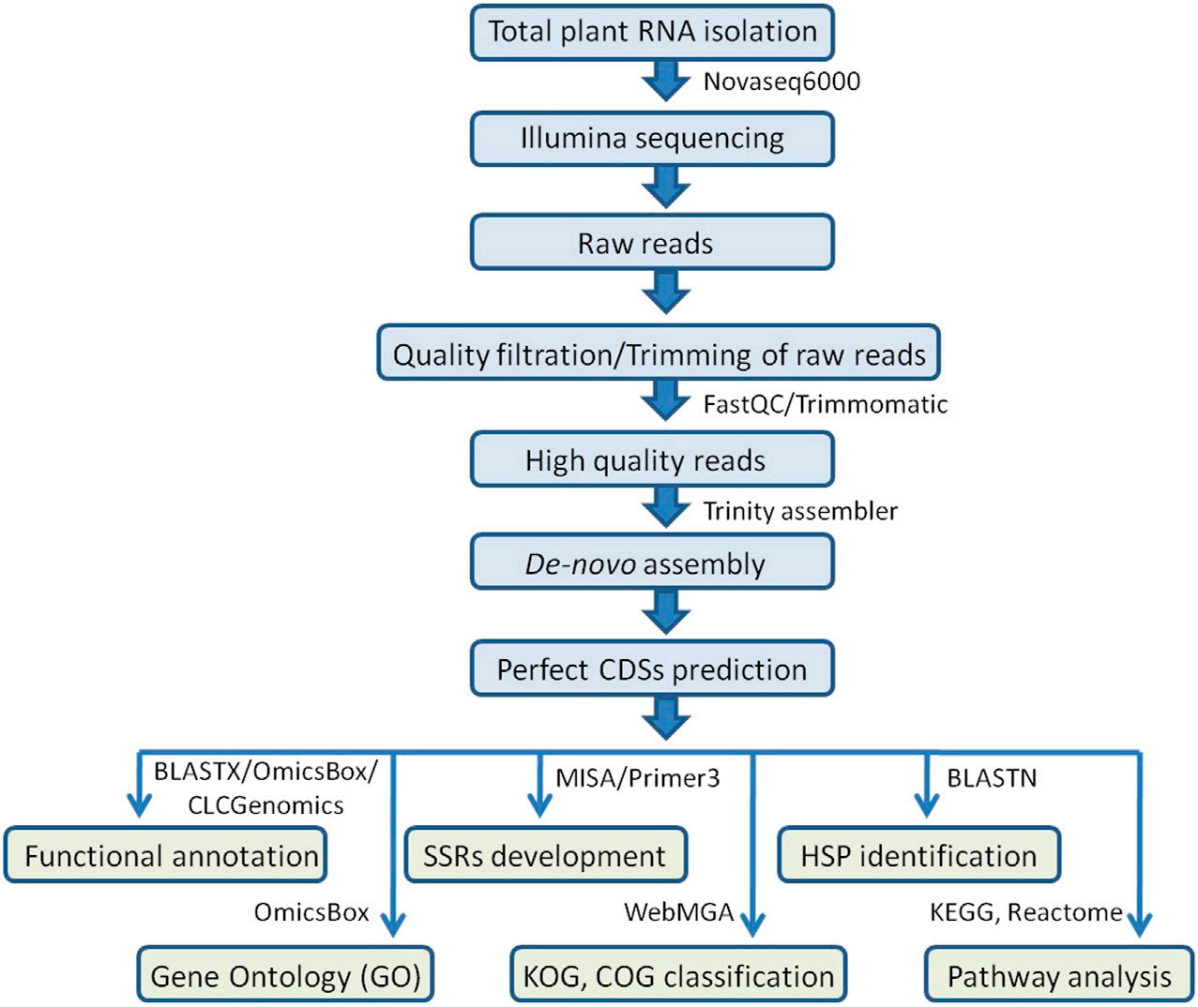
Workflow for Illumina sequencing, *de-novo* transcriptome assembly, functional annotations, SSR development, and identification of HSPs carried out in the allohexaploid Brassica.

The MIcroSAtellite (MISA) identification tool Perl script (http://pgrc.ipk-gatersleben.de/misa/misa.html) was used to find probable microsatellite loci in coding sequences extracted from *de-novo* assembled transcripts. The parameters were adjusted to find perfect mono-, di-, tri-, tetra-, penta-, and hexa-nucleotide motifs with a minimum number of 10, 6, 5, 5, 5, and 5 repetitions, respectively. Primer3 v0.4.0 was used to create the primer pairs. For primer pair designing, the following parameters were used: PCR product size: 100–400 bp (optimum: 280 bp), GC content: 45–70% (optimum: 50%), Tm: 57–62°C (optimum: 60°C), primer size: 18–25 bp (optimum: 20 bp), GC content: 45–70% (optimum: 50%), and Tm: 57–62°C (optimum: 60°C) (Figure 1).

## Results

### *De-novo* transcriptome assembly

**FIGURE 2:**
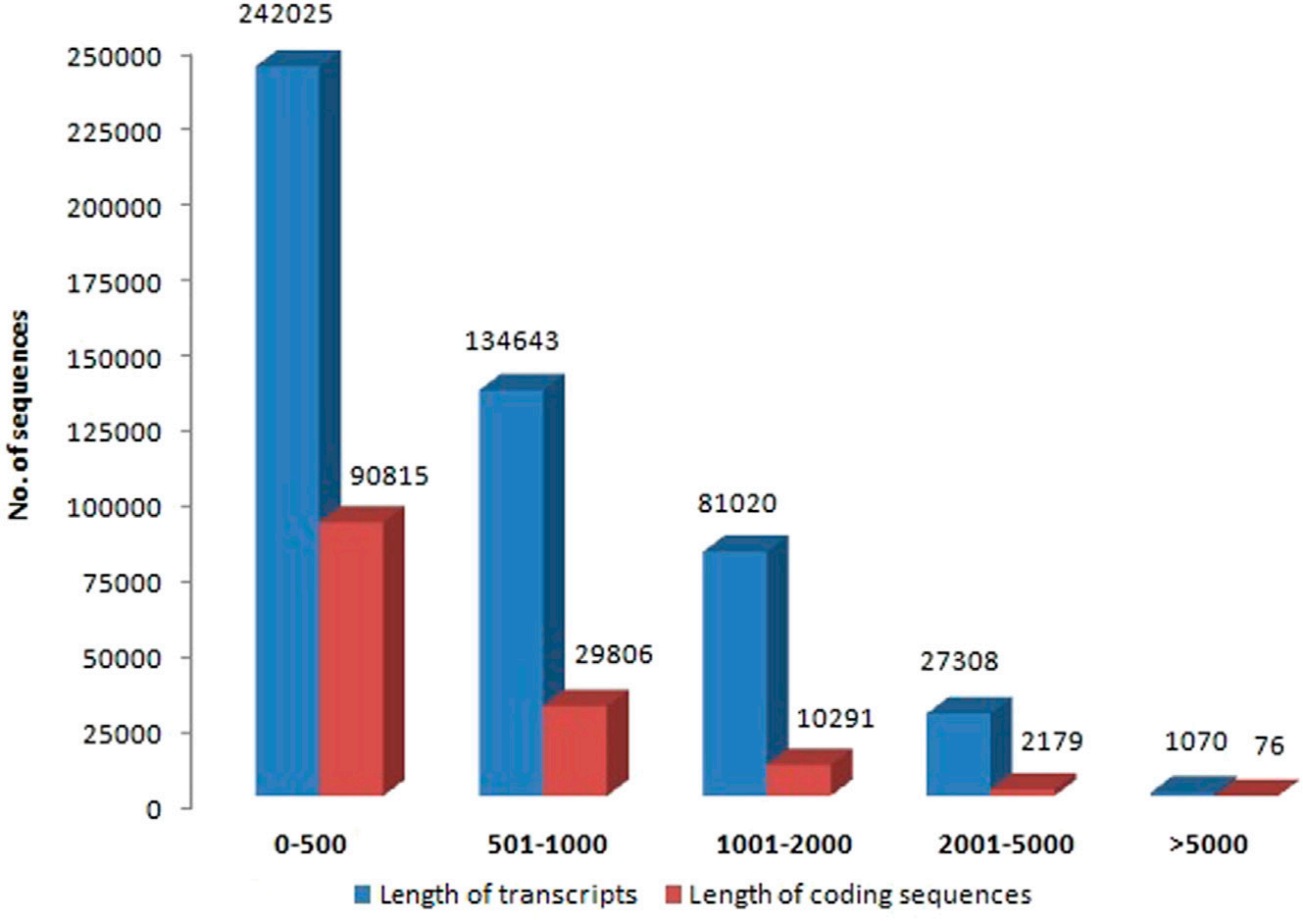
Length distribution of allohexaploid Brassica transcripts and coding sequences.

**FIGURE 3:**
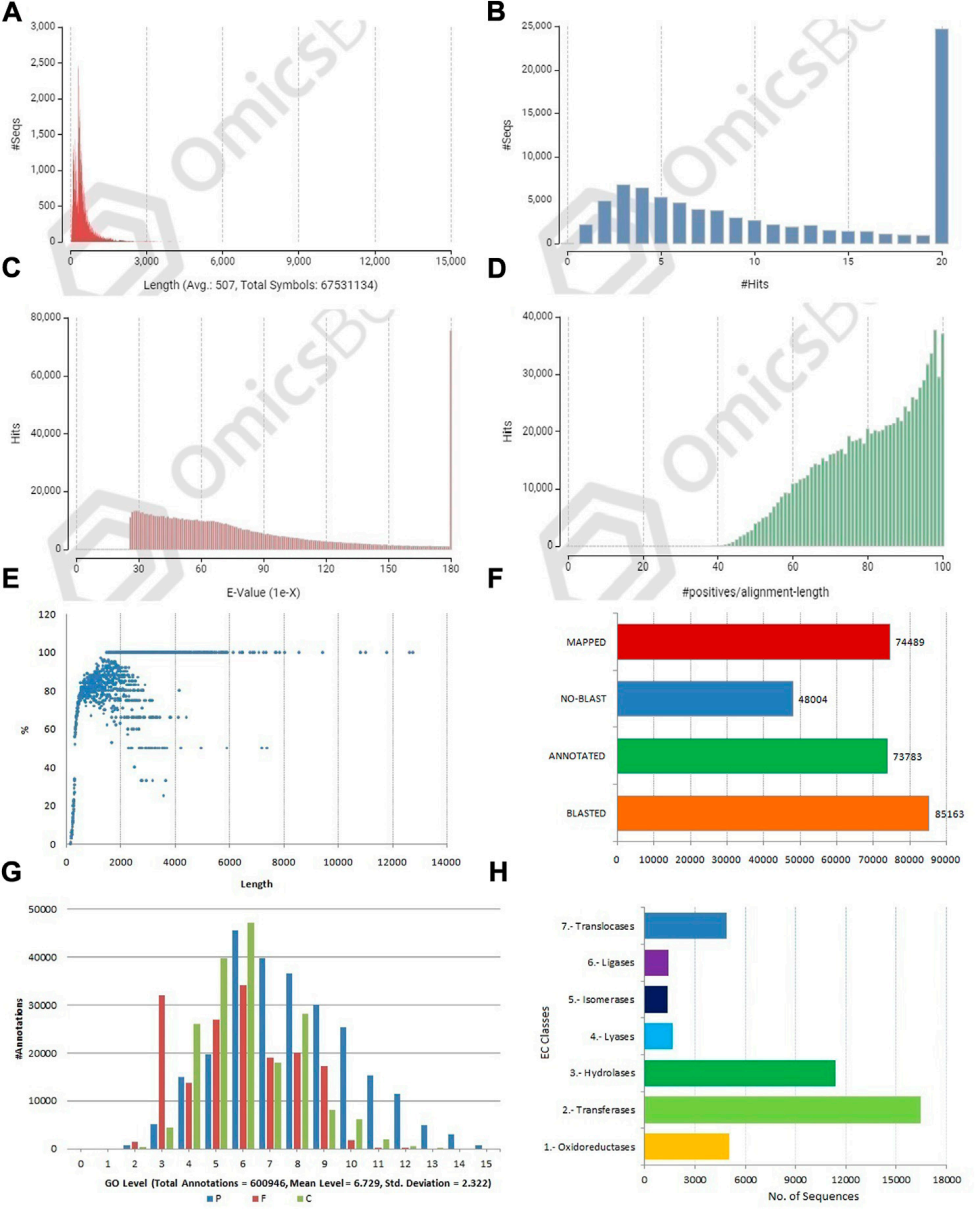
Structural and functional annotation of allohexaploid transcriptome assembly. **(A)** Sequence length distribution of coding sequences; **(B)** BLASTX hit distribution; **(C)** E-value distribution; **(D)** Sequence similarity distribution of BLASTX hits; **(E)** Annotation percent of coding sequences against length. **(F)** Three-step distribution of OmicsBox process (BLASTX, mapping, and annotation); **(G)** GO level distribution for biological process, molecular function, and cellular component [P-BP, F-MF, and C-CC]; **(H)** Enzyme code distribution for major classes.

Illumina Novaseq 6000 runs generated a total of 76,055,546 raw paired-end reads, including low-quality sequences, adapter-primer sequences, and very short reads. This was accomplished using cDNA generated from the total mRNA of allohexaploid Brassica. The raw reads showed a total GC content of 44.94% and a mean sequence length of 151 bp. At a Phred score (Q) of 30, low-quality bases, adapter-primer sequences, and very short sequences were eliminated. A total of 74,693,278 high-quality clean reads were obtained after a thorough quality check and data filtering, with 11,012,782 reads (98.01%) with Q20 and 10,583,124 reads (94.19%) with Q30. With 44.93% GC content, the clean reads totaled 11,236,399 kb in length. The BUSCO of assembly showed 93.3% completeness for conserved ortholog content. The raw reads (Accession No. SRR14934389) were submitted to the Sequence Read Archive (SRA) database at the National Center for Biotechnology Information (NCBI) in the United States. There were 486,066 transcripts generated from the paired-end reads, with a total length of 368,936,034 bp and an average length of 759.02 bp per transcript. The transcripts N50 and N70 values were 1,069 and 627 bp, respectively, with the longest transcript length of 33,251 bp. The transcripts could be divided into five categories based on their length: 1) 242,025 transcripts (49.79%) with a length of less than 500 bp; 2) 134,643 transcripts (27.7%) with a length range of 501–1,000 bp; 3) 81,020 transcripts (16.67%) with a length range of 1,001–2000 bp; 4) 27,308 transcripts (5.62%) with a length range of 2001–5000 bp; and 5) only 1,070 transcripts (0.22%) with length >5000 bp (Figure 2). From these transcripts, 133,167 coding sequences (CDSs) were predicted, with a total length of 67,531,134 bp and an average length of 507.12 bp, with a range of 93–14,688 bp. CDSs had a GC content of 46.15%, and their N50 and N70 values were 606 bp and 417 bp, respectively. Furthermore, 90,815 CDSs (68.2%) had lengths less than 500 bp; 29,806 CDSs (22.38%) had lengths between 501 and 1,000 bp; 10,291 CDSs (7.73%) had lengths between 1,001–2,000 bp; 2,179 CDSs (1.64%) had lengths between 2,001 and 5,000 bp; and 76 CDSs (0.06%) had lengths greater than 5,000 bp (Figure 2). The statistics of *de-novo* assembly and CDSs are given in Table. 1. The length distribution of CDSs is shown in Figure 3A.

**TABLE 1:** Summary of results obtained after the development of *de-novo* assembly of allohexaploid Brassica transcriptomes.

Parameters	Transcripts	CDSs
Sum bp	368936034	67531134
Number of sequences	486,066	133,167
Average	759.02	507.12
Largest sequence	33,251	14,688
N50	1,069 (99,977)	606 (30,386)
N60	824 (139312)	498 (42,738)
N70	627 (190765)	417 (57,616)
N80	473 (258626)	351 (75,274)
N90	343 (350035)	297 (96,113)
N100	173 (486066)	93 (133167)
N_count	0	0
Gaps	0	0

### Functional annotations of coding sequences

**FIGURE 4:**
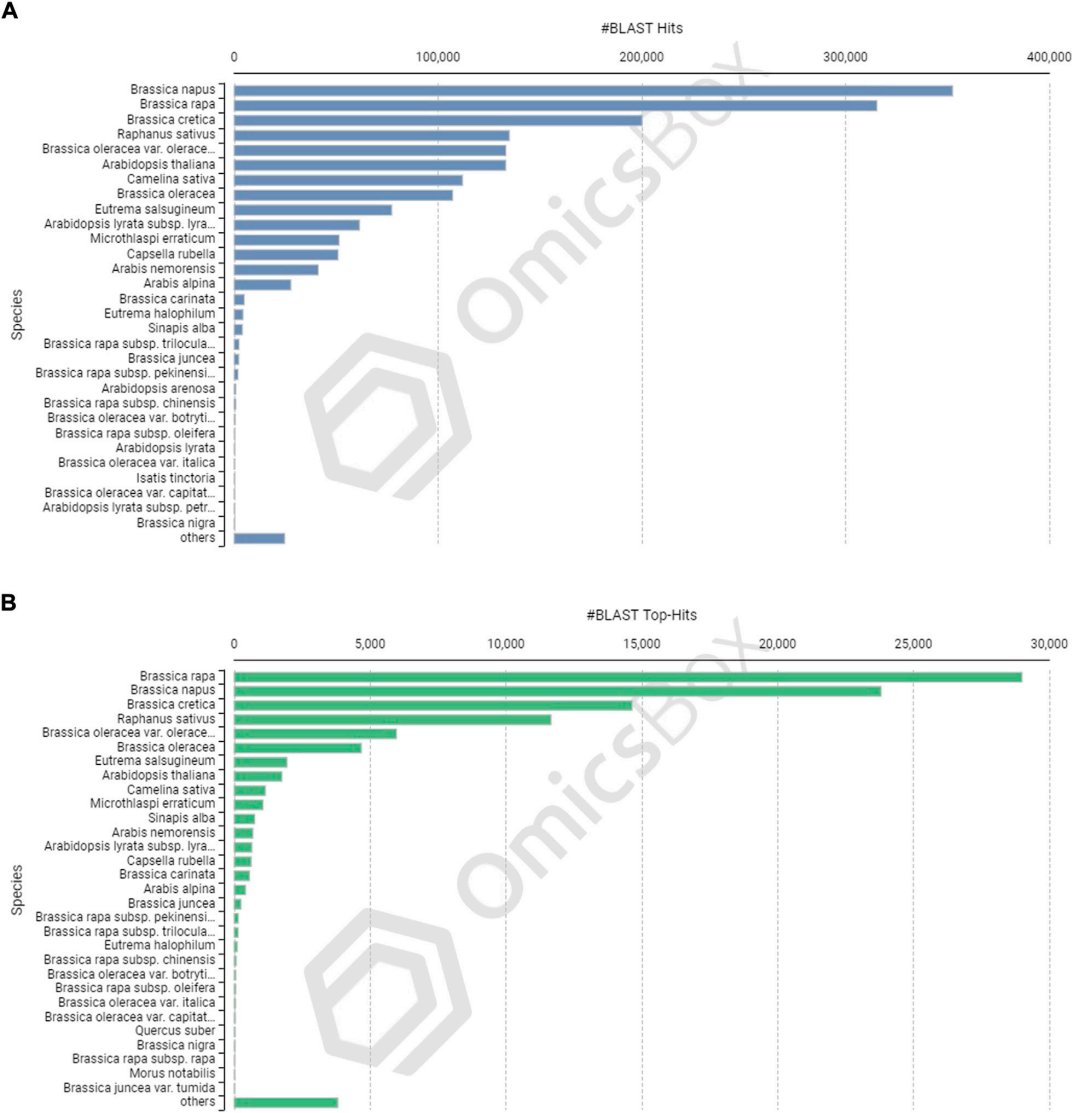
Species-based distribution of the BLASTX hits **(A)** and Top BLASTX hits **(B)** for each coding sequence of allohexaploid Brassica.

The CDSs were masked for repetitions by RMBlast search engine using Dfam v3.0 consensus database using Brassicaceae as species filter with OmicsBox tool. A total of 880,817 bp (1.30%) were masked by the search engine, with 1.0% belonging to simple repeats and 0.3% to low complexity sequences. The masked CDSs were used for annotation, and probable functions were assigned to them. The CDSs were locally BLASTX at an E value of 1e-25 against the reference protein sequences of *A. thaliana* and *B. rapa*. Between the query and the local protein database, the blast search found 85,163 (63.95%) unique hits. The remaining CDSs, on the other hand, showed no resemblance to the database (Figures 3B,F). CDS similarity was calculated using E-values ranging from 1e-26 to 1e-180 (Figure 3C). The sequence similarity distribution of BLASTX hits ranged from 36 to 100%, with the largest number of hits (37,672) falling in the 98% range. In 37,005 BLAST hits, there was an absolute similarity between query and database sequences. However, 56.16% of the hits had a resemblance of more than 80%, whereas only 43.84% of the hits had a similarity of 35%–80% (Figure 3D). The smallest CDS (153 bp) had just 1% annotation, whilst the largest CDS (12,741 bp) had 100% annotation (Figure 3E). A total of 74,489 and 73,783 CDSs were mapped and annotated with GO terms, respectively, according to the tag distribution analysis (Figure 3F). The CDSs had a top-hit similarity of more than 99% with Brassicaceae species, indicating that our transcriptome assembly had a reasonable coverage of homologous sequences. Based on BLASTX results, 600,946 GO level annotations were recorded in the biological process, molecular function, and cellular components categories. Only one GO term was found in a large percentage of CDSs, and the maximum number of GO terms (6,251) was found in CDSs with a length of 315 bp (Figure 3G). There were 42,225 CDSs identified and categorized into seven enzyme coding classes (Figure 3H). The BLASTX program was used to find homology between CDSs of allohexaploid Brassica and CDSs from other related species in the NCBI Nr database, and it revealed that many plant species in the Brassicaceae family are related (Figures 4A and B).

### Gene ontology classification

**FIGURE 5:**
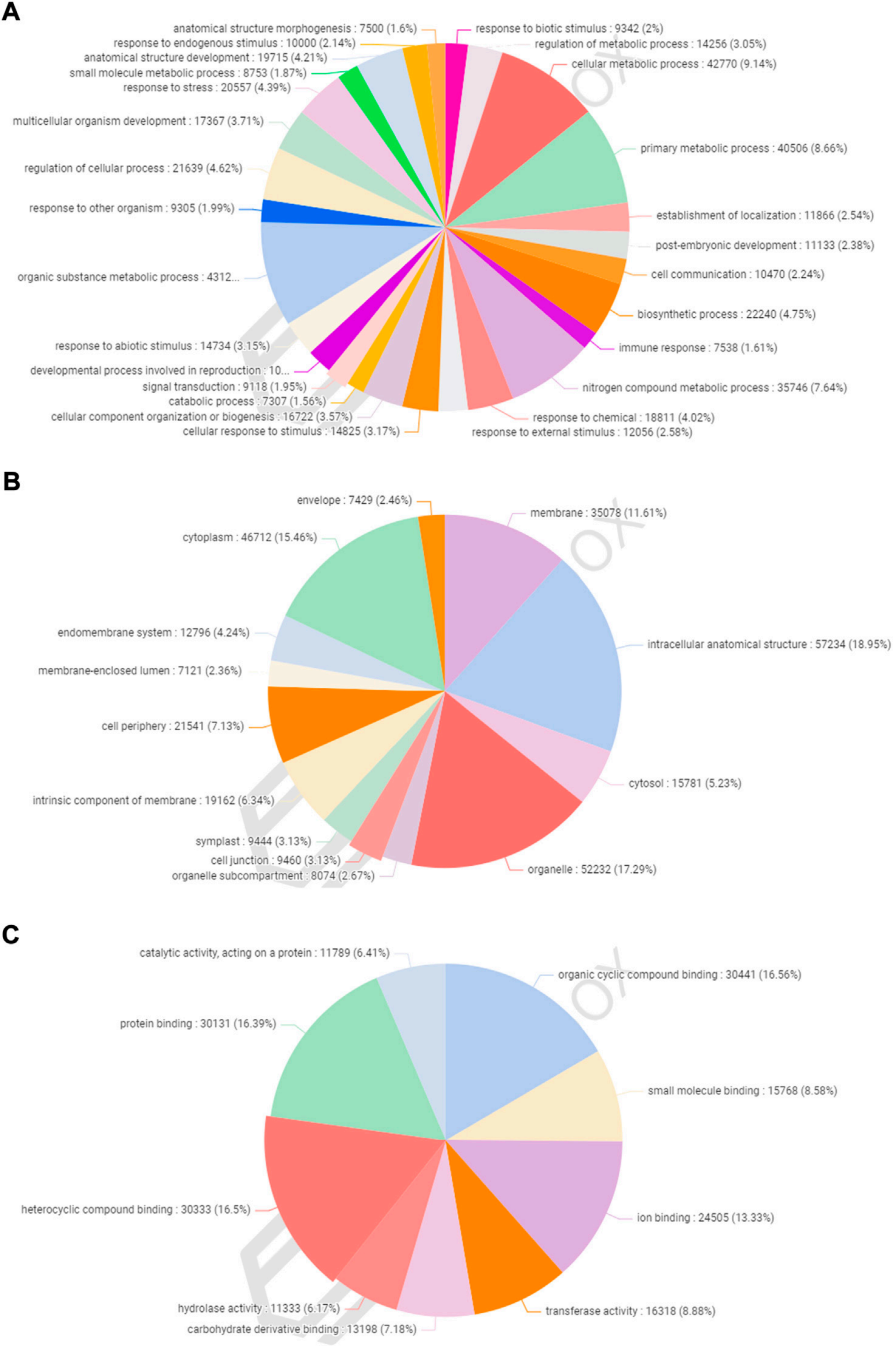
Functional classification of gene ontology (GO) terms under biological process **(A)**, cellular component **(B)**, and molecular function **(C)** categories.

A total of 77,613 (58.28%) CDSs were assigned to gene ontology terms with a maximum and minimum of 63 and one GO IDs, respectively. A BLASTX search was used to classify the CDSs of allohexaploid Brassica into three functional categories (biological processes, cellular components, and molecular functions). All CDSs that fall under various functional categories were allocated a total of 953,773 GO terms. Biological processes were assigned the greatest number of GO terms (467,893; 49.06%), followed by cellular components (302,064; 31.67%), and molecular functions (183,816; 19.27%). Among the biological processes, organic substance metabolism (43,122; 9.22%) had the highest number of CDSs, followed by cellular metabolism (42,770; 9.14%), primary metabolism (40,506; 8.66%), nitrogen compound metabolism (35,746; 7.64%), biosynthetic process (22,240; 4.75%), and cellular process regulation (21,639; 4.62%). (Figure 5A). In the cellular component category, CDSs in the intracellular anatomical structure (57,234; 18.95%) were the largest group, followed by organelle (52,232; 17.29%), cytoplasm (46,712; 15.46%), and membrane (35,078; 11.61%) (Figure 5B). The CDSs involved in organic cyclic compound binding (30,441; 16.56%) were the most numerous among the molecular functions, followed by heterocyclic compound binding (30,333; 16.50%), protein binding (30,131; 16.39%), and ion binding (24,505; 13.33%). (Figure 5C).

### Functional classification of CDSs by protein databases

To obtain the corresponding annotation information, all of the CDSs were BLAST against various protein databases, including Nr, Swiss-Prot, Refseq, COG, KOG, Pfam, Prk, and Tigrfam. A total of 87,781 CDSs (65.92%) were aligned to the Nr database, with about 59,413 CDSs (67.68%) showing more than 90% similarity. The CDSs that corresponded with the database in the case of the Refseq protein database yielded a total of 86,226 positive hits. Furthermore, using CLC genomics workbench, all CDSs were aligned against the Swiss-Prot protein database at an E-value of 1e-25. In CDSs, a total of 126,131 (92.46%) positive hits were found, with 27,704 CDSs sharing more than 90% similarity. Furthermore, BLASTP was performed on these CDSs against the Pfam, Prk, and Tigrfam protein databases, yielding 120,489, 36,476, and 39,368 positive hits, respectively.

**FIGURE 6:**
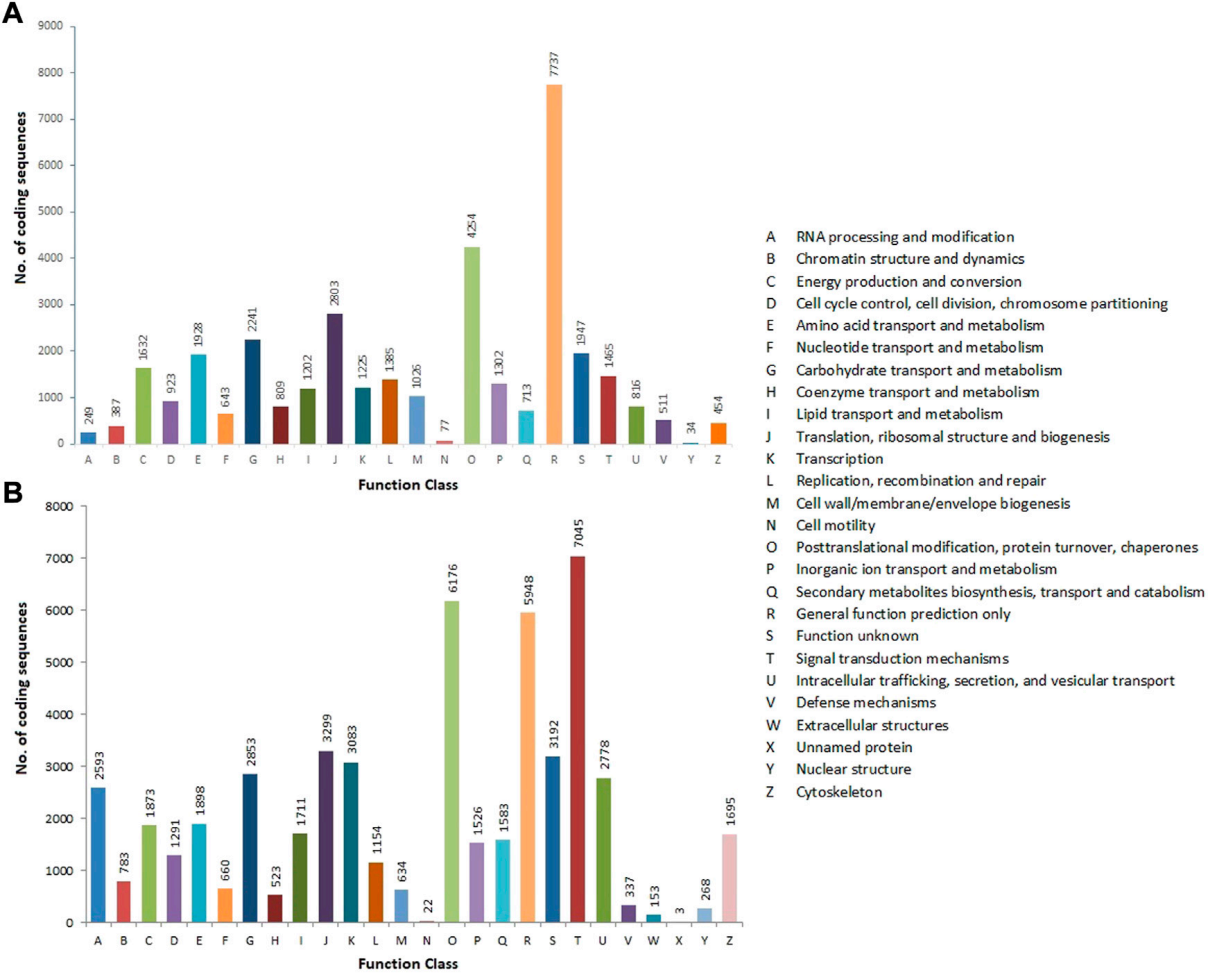
COG **(A)** and KOG **(B)** functional classification of allohexaploid Brassica coding sequences. The *y*-axis indicates the number of coding sequences in a specific functional cluster. The *x*-axis indicates the functional classes.

The CDSs were annotated against various protein databases, such as COG for prokaryotes and KOG for eukaryotes, using the RPSBLAST software, which was run over the WebMGA web server, with an E-value of 1e-25. In total, 35,763 CDSs were assigned to the COG database’s 24 functional classes. General functions (7,737; 21.63%), followed by posttranslational modification, protein turnover, and chaperones (4,254; 11.89%), translation, ribosomal structure, and biogenesis (2,803; 7.84%), carbohydrate transport and metabolism (2,241; 6.26%), unknown function (1,947; 5.45%), and nuclear structure (34; 0.09%) were the classes with the most CDSs (Figure 6A). The KOG database was used to functionally annotate 53,081 CDSs and divide them into 26 types. Signal transduction mechanisms had the largest cluster (7,045; 13.27%), followed by posttranslational modification, protein turnover, and chaperones (6,176; 11.64%), general function (5,948; 11.2%), translation, ribosomal structure, and biogenesis (3,299; 6.21%), unknown function (3,192; 6.01%), transcription (3,083; 5.81%), and unnamed proteins (3; 0.01%) (Figure 6B) (Table 2) (Supplementary Table S1).

**TABLE 2:** BLAST hit percentage of annotated coding sequences in different protein databases.

Value	Total	Nr	Swiss-Prot	Refseq	COG	KOG	KEGG	GO	Pfam	Prk	Tigrfam	Average
No.	133,167	87,781	126,131	86,226	35,763	53,081	29,810	77,613	120,489	36,476	39,368	69,273.8
%	100	65.92	94.72	64.75	26.86	39.86	22.39	58.28	90.48	27.39	29.56	52.02

### Functional classification by Kyoto Encyclopedia of Genes and Genomes and Reactome pathways

**FIGURE 7:**
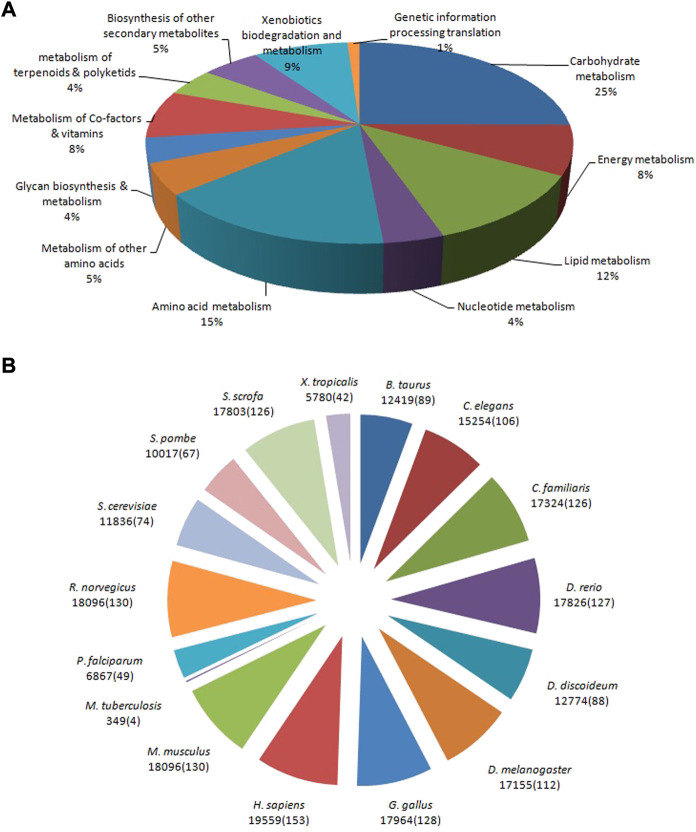
Pathway assignment based on the Kyoto Encyclopedia of Genes and Genomes: KEGG **(A)** and Reactome **(B)** databases.

The allohexaploid Brassica CDSs were examined further in the KEGG and Reactome pathway databases. A total of 29,810 (22.38%) significant matches in the database were assigned to 144 KEGG pathways, including the metabolism and genetic information processing translation, out of a total of 133,167 CDSs. In the metabolism category, carbohydrate metabolism (7,481; 25%) was the most common sub-category, followed by amino acid metabolism (4,594; 15%), lipid metabolism (3,461; 12%), xenobiotic biodegradation and metabolism (2,568; 9%), energy metabolism (2,370; 8%), metabolism of cofactors and vitamins (2,302; 8%), biosynthesis of other secondary metabolites (1,563; 5%), and nucleotide metabolism (3,461; 12%). (1,148; 4%). A single aminoacyl-tRNA biosynthesis pathway with 325 CDSs was discovered under the genetic information processing translation category (Figure 7A). *Homo sapiens* (Human) had the most pathways in the Reactome database (153; 9.87%), followed by *Mus musculus* (House mouse) (130; 8.38%), *Rattus norvegicus* (Brown rat) (130; 8.38%), *Gallus gallus* (Fowl) (128; 8.26%), *Danio rerio* (Zebrafish) (127; 8.19%), and *Canis familiaris* (Dog) (126; (4; 0.26%). Due to their close proximity, *M. musculus* and *R. norvegicus* demonstrated a total of 18,096 CDSs belonging to 130 pathways. However, in contrast, 126 pathways were identified to belong to *C. familiaris* and *S. scrofa* with 17,324 and 17,803 CDSs, respectively (Figure 7B) (Supplementary Table S2).

### Genes putatively related to heat stress tolerance

The downloaded HSPs are 13,279,051 bp in length, with an average size of 1,019.74 bp. HSPs had the greatest and smallest gene sizes of 11,682 and 30 bp, respectively, with an N50 value of 1,413 bp (*n* = 3,240). BLASTN results indicated a total of 2,012 CDSs in allohexaploid Brassica, with over 95% similarity with all of the plants’ downloaded HSPs. However, the vast majority of CDSs (99.85%) share absolute homology with these HSPs. The 100% similarity between CDSs and HSPs further confirmed the highly conserved character of these proteins across plant taxa. The molecular weights of the downloaded HSPs that showed similarity with CDSs ranged from 14.7 to 70 kDa. The bulk of HSPs, however, belonged to the small HSP category, with MW ranging from 14.7 to 26.5 kDa. These HSPs show up in a variety of cell sites, such as the cytosol, peroxisome, chloroplast, and mitochondria. The sHSPs were categorized into class I–class VI categories based on their involvement in the cell, out of the total discovered HSPs of allohexaploid (Supplementary Table S3).

### SSRs identification and development

**FIGURE 8:**
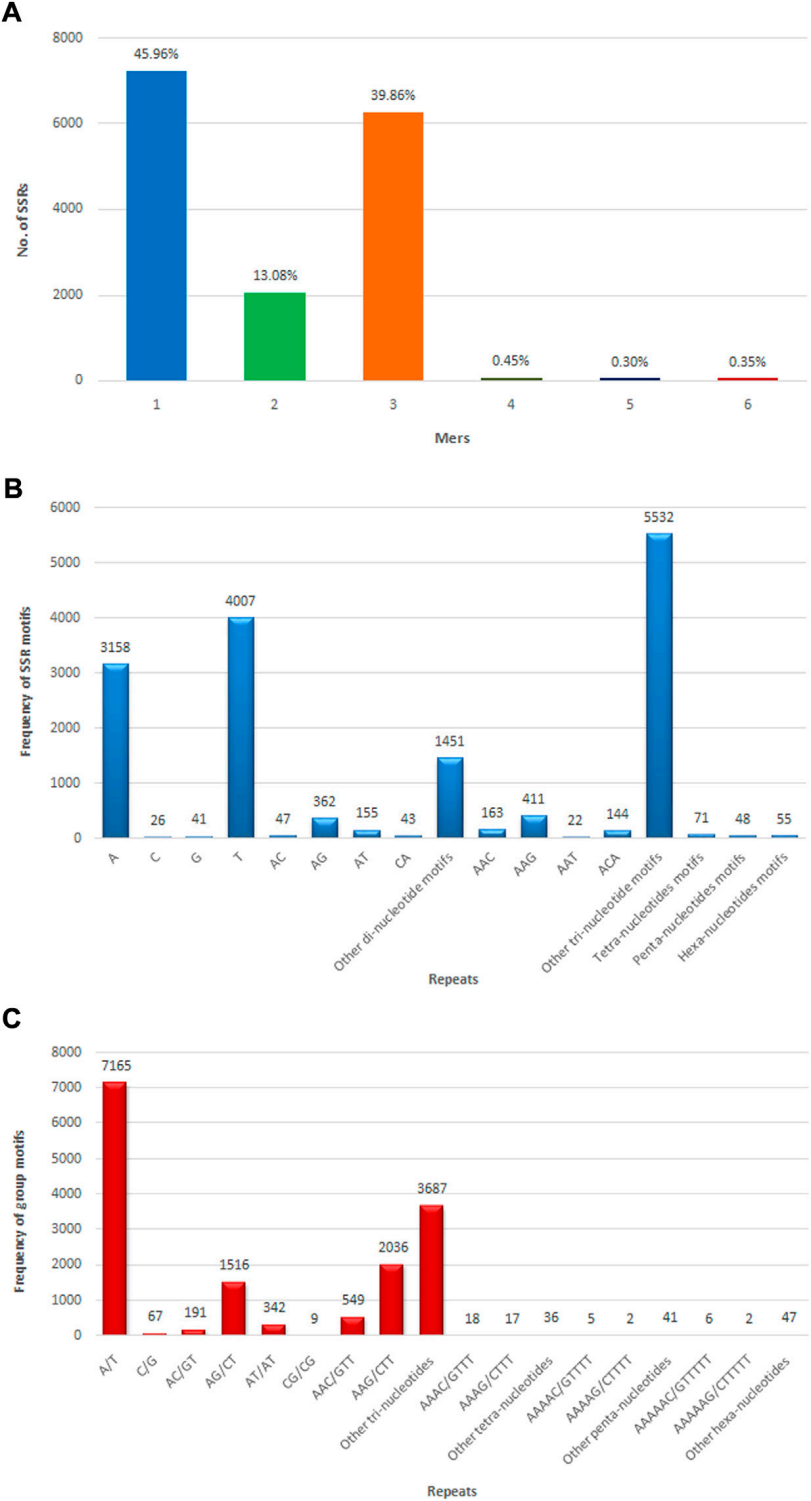
Numbers of different types of SSRs identified in allohexaploid Brassica transcriptomes. **(A)** Different repeat motif distribution in total SSRs; **(B)** Top motif distribution in SSRs; **(C)** Abundance of different grouped repeat motifs in total SSRs.

The SSR prediction and primer designing were performed in CDSs of assembly developed using MISA and Primer3 software. MISA has analyzed a total of 133,167 CDSs for SSR prediction, but only 13,595 sequences were found to have SSRs. From 133,167 CDSs, 15,736 potential SSRs were identified, with 1,814 CDSs having multiple microsatellite markers. There were 1,407 compound microsatellite markers among the 15,736 SSRs. Microsatellite markers were detected in 11.82% of allohexaploid Brassica CDSs, with an average of one SSR every 4.29 kb of CDS length. Mononucleotide repeats were detected in the highest number of microsatellites (7,232 or 45.96%), followed by di-, tri-, tetra-, penta-, and hexa-nucleotide repeats in 2,058 (13.08%), 6,272 (39.86%), 71 (0.45%), 48 (0.30%), and 55 SSRs (0.35%), respectively (Figure 8A). The most common mononucleotide motif was T, which was discovered in 4,007 (25.46%) SSRs, followed by A, G, and C, which were found in 3,158 (20.07%), 41 (0.26%), and 26 (0.16%) SSRs, respectively. TC (496; 3.15%), CT (424; 2.69%), AG (362; 2.30%), GA (234; 1.49%), TA (187; 1.19%), AT (155; 0.99%), TG (68; 0.43%), AC (47; 0.30%), CA (43; 0.27%), GT (33; 0.21%), CG (5), and GC (4) were the most common dinucleotide motifs in SSRs. The trinucleotide motifs were found in 60 different combinations, with GAA (627; 3.99%) being the most common, followed by AAG (411; 2.61%), AGA (316; 2.01%), TCT (302; 1.92%), and TTA and TAG (9) being the least common. There were 71, 48, and 55 microsatellites with tetra-, penta-, and hexa-nucleotide motifs, respectively (Figure 8B).

The most abundant grouped motif in the mononucleotide repeats was A/T (7,165; 45.53%), followed by C/G (67; 0.42%). Among the dinucleotide repeats, the most frequent repeat motif was AG/CT (1,516; 9.63%), followed by AT/AT (342; 2.17%), AC/GT (191; 1.21%), and CG/CG (9; 0.06%). Among the trinucleotide repeat motifs, the most abundant repeat motifs was AAG/CTT (2,036; 12.94%), followed by AGG/CTT (1,165; 7.40%), ATC/ATG (988; 6.28%), ACC/GGT (564; 3.58%), AAC/GTT (549; 3.49%), AGC/CTG (464; 2.95%), CCG/CGG (195; 1.24%), ACG/CGT (120; 0.76%), ACT/AGT (99; 0.63%), and AAT/ATT (92; 0.58%). The tetra-, penta-, and hexa-nucleotide grouped motifs were present in 71 (0.45%), 48(0.30%), and 55 (0.35%) microsatellites, respectively (Figure 8C) (Supplementary Table S1). The details included in the table for microsatellites are unique ID, positions of microsatellite flanking regions in the CDSs, sequence, length of forward and reverse primers, melting temperature, and expected PCR product size (Supplementary Table S4).

## Discussion

The cultivated members of Brassicaceae existing in nature are either diploid (*B. rapa*, *B. nigra*, *B. oleracea*) or tetraploid/amphidiploids (*B. junea*, *B. napus*, *B. carinata*) (Yang et al., 2016). However, due to a considerable benefit over existing cultivated Brassica, such as combining genetic diversity and traits from all three crops and related genomes, which leads to allelic heterosis from additional genomes, various attempts have been made to generate an allohexaploid Brassica. As a result, the Brassicaceae family has been extensively studied for developing synthetic polyploids (hexaploids) using cultivated and wild members *via* embryo rescue, followed by chromosome doubling *via* colchicine treatment (Meng et al., 1998; Rahman, 2001; Pradhan et al., 2010; Gupta et al., 2016; Mwathi et al., 2017; Mwathi et al., 2020) and protoplast fusion used as an alternative approach for overcoming from pre- and post-fertilization barriers (Primard et al., 1988; Lelivelt et al., 1993; Gaikwad et al., 1996; Wang et al., 2005). However, in previously established hexaploid Brassicas, genome stability was a serious concern; therefore, they were either sterile or aneuploids (Mason et al., 2012; Mwathi et al., 2019). Polyploids enhanced the gene pool of cultivated plant species, allowing for better tolerance to environmental and biological challenges and the production of novel allelic gains (Osborn, 2004; Hu et al., 2019; Yin et al., 2020). When *B. juncea* is stressed by heat or dryness, the seed set is severely impeded (Anand et al., 2010; Youssefi et al., 2011). The novel allohexaploid (H1; *B. juncea* + *S. alba*) is promising for the future due to its high temperature tolerance of up to 40.2°C during seed developmental stages, as well as resistance to two major diseases, including *Alternaria brassicae* blight, which is not present in all cultivated Brassica varieties (Kumari et al., 2018). Apart from them, due to the restoration of regular meiosis and amphidiploid brassicas, the unique allohexaploid (H1) is possible. Because of its crossability with cultivated diploid and allotetraploid Brassicas, it is considered an “elite” breeding material. Polyploid plants are more tolerant to abiotic stresses than their diploid counterparts (Saleh et al., 2008; Chandra and Dubey, 2010; Van et al., 2011; Manzaneda et al., 2012). Stress-related genes are preserved during the polyploidization process in *Arabidopsis thaliana*, according to a genome-wide investigation of gene expression (Casneuf et al., 2006). The number of CDSs discovered from transcripts in this study was larger than the total number of unigenes identified in *B. juncea* (77,750) and *S. alba* (47,972), the parents of the H1 allohexaploid (Bhardwaj et al., 2015; Zhang et al., 2016). Furthermore, the GC percentage of CDSs was greater than the average GC percent of transcriptome assemblies from *B. juncea* (41.92) and *S. alba* (46.63). (Sinha et al., 2015; Zhang et al., 2016). Due to the lack of a reference transcriptome assembly to assess the quality of CDSs, the N50 value was employed instead, which was found to be adequate (Mundry et al., 2012). The *de-novo* transcriptome assembly was annotated against protein databases (Nr, Swiss-Prot, Refseq, COG, KOG, KEGG, GO, Pfam, Prk, and Tigrfam) to provide a comprehensive result for genetic investigation. The protein-coding potential of sequences was confirmed by a maximum of about 94% positive hits of allohexaploid CDSs against the public protein database. Due to incomplete sequences or inadequate information on the *S. alba*, *B. juncea*, and related species in the databases, certain genes cannot be linked with any functional annotation. Unmatched sequences, on the other hand, are nevertheless a valuable resource for allohexaploid use. To determine the evolutionary link, the CDSs of allohexaploid plants revealed a high level of absolute sequence similarity with Brassicaceae plant species, indicating that the assemblies are homologues. Similarly, *Cyamopsis tetragonoloba* (Fabaceae) has the strongest phylogenetic relationship to leguminous plants in a transcriptome analysis (Rawal et al., 2017; Tanwar et al., 2017; Qurainy et al., 2019).

The GO terms are a valuable resource for determining gene function (Ashburner et al., 2000). A total of 77,613 CDSs have been assigned to the 953,773 GO terms that have been divided into three functional classes (biological process, cellular components, and molecular function). Many GO terms (e.g., response to abiotic and biotic stimulus, immune response, stress response, and response to other organisms) have substantial significance to allohexaploid’s wide pathogen tolerance and high temperature and present new avenues for future research. The allohexaploid brassica (H1) and *S. alba* (one of their parents) have been reported to be resistant to a variety of fungal diseases, insect pest, drought, and heat tolerance (Kumari et al., 2018; Kumari et al., 2020a; Kumari et al., 2020c). The genetic mechanism underlying all of these resistance responses, however, is still unknown. We used the RPSBLAST tool to annotate CDSs against the COG and KOG protein databases using the WebMGA web server. KOG identifiers (IDs) are used to identify orthologous and paralogous proteins among eukaryotes that have assigned roles to novel genes (Liu et al., 2016). In the COG and KOG databases, a total of 26.86% and 39.86% of CDSs were classified into 24 and 26 functional classes, respectively. A larger number of COG and KOG classifications revealed that the CDSs in allohexaploid Brassica comprise a diverse set of genes. Many functional classes linked with allohexaploid stress responses were identified in these databases, including defense mechanisms, chaperons, secondary metabolite production, and glucose transport and metabolism (Lopez et al., 2017). The Kyoto Encyclopedia of Genes and Genomes (KEGG) is a curated database for gene functional annotations, with the results displayed in the form of graphics of various biochemical pathways and an additional table of orthologous groups that depicted the evolutionary link between genes (Ogata et al., 1999). In the current study, the majority (98.9%) of total CDSs (29,810) assigned to KEGG were found to be connected to metabolic pathways. Antibiotic, Brassinosteroid, starch and sucrose metabolism, insect hormone, cutin, suberin and wax biosynthesis, glucosinolate, and secondary metabolite biosynthesis were all detected in the allohexaploid Brassica CDSs and were putatively associated with the wide range of adaptations during harsh stress conditions (Beck et al., 2007; Peng et al., 2015; Zielniok et al., 2015; Kumari et al., 2020a). Reactome is an open-source pathway database for curated animal proteins that is freely accessible. NCBI, Ensembl, UniProt, KEGG (Gene and Compound), ChEBI, PubMed, and GO are widely used to cross-reference the content of this database. The Reactome database graphically depicts the enriched pathways (Cai et al., 2020).

HSPs are implicated in disease and insect pest invasion and stress conditions such as heat, alkalinity, drought, low temperature, and UV light (Boston et al., 1996; Bhattarai et al., 2007; Breiman, 2014). Resistant protein stability, immunity regulation, and programmed cell death are all regulated by the HSP70 and sHSP families (Kim et al., 2007; Ooijen et al., 2010; Liu and Whitham, 2013). Protein homeostasis, or maintaining appropriate concentration, conformation, and subcellular location of proteins in the cytosol, peroxisome, chloroplast, and mitochondria, was found to be aided by these sHSP (Hoppe and Cohen, 2020). Restricted tobacco etch potyvirus (TEV) mobility, enhanced DNA methylation, an ATP-independent chaperone that prevents protein aggregation and protects against cell stresses, serves as co-chaperones, and other molecular roles were assigned to these HSPs. HSPs with MW greater than 20.0 kDa were found in chloroplasts or mitochondria, while those with MW less than 20.0 kDa were found in peroxisomes. HSPs with molecular weights of 57 and 70 kDa were found in the cytosol. The presence of HSPs in the cytosol confers resistance to biotic and abiotic stresses (Vierling, 1991; Boston et al., 1996). HSP70 in the cytosol is required for the hypersensitive response (HR) to pathogen attack and non-host resistance (Kanzaki et al., 2003). Gao et al. (2021) provided heat shock treatment at 40 and 60°C to analyze gene expression patterns for the high temperature tolerance in *B. napus*. The DEGs examination study identified a total of 442 genes in seeds treated with high temperature that belongs to posttranslational modifications, protein turnover, chaperons, carbohydrate transport, metabolic pathways, and secondary metabolite biosynthesis pathways. Out of all these DEGs, they have identified only 6 sHSP and 22 transcription factor genes that were involved in heat stress tolerance. Wang et al. (2018b) conducted a study on *Lentinula edodes* by transcriptome and proteome analysis to identify proteins related to thermotolerance. They have identified various types of heat shock proteins, such as HSP40, HSP70, and HSP90, in association with tryptophan and IAA pathways. Yu et al. (2014) identified genes that worked under heat-stress conditions at the time of seed development in *B. napus* through transcriptome profiling. The study of DEGs identified many upregulated genes, including heat transcription factors (13 HSFs), heat shock proteins (91 HSPs; DnaJ/Hsp40, Hsp60/10, Hsp70, Hsp90, Hsp101, and sHsp), and heat-related marker genes such as ROF2, DREEB2a, MBF1c, and Hsa32. Wang et al. (2016) used Chinese cabbage (*Brassica rapa* ssp. chinensis) strains for comparative transcriptional analysis to reveal the heat-responsive genes. Out of 625 DEGs identified in their study, two HSPs, i.e., Bra034104 (HSP70-1) and Bra030036 (HSP) with some genes from the WRKY gene family (Bra015372, WRKY7; Bra017561, WRKY8, and Bra006178, WRKY75) were reported to be expressed under high temperature conditions. Guo et al. (2019) identified a total of 23 heat shock transcription factors and 61 heat shock proteins (HSP100/ClpB, HSP90, HSP70, and sHSPs) that were upregulated upon the heat treatment of four different strains of Chinese kale (*Brassica alboglabra*). Moreover, Lohani et al. (2021) used comparative transcriptome profiling of heat stressed and non-stressed pollen and pistil (stigma and style) to identify DEGs for heat tolerance in *B. napus*. They have identified a variety of proteins involved in heat tolerance, i.e., heat stress transcription factors (HSFs) and heat shock proteins (HSPs)/chaperons. They have identified small heat shock proteins such as 15.7, 17.6, 17.8, 18.5, 21, 22, 23.6, 26.5, and 70 kDA in pollen and pistil, as we recorded in our studies. The presence of a diverse set of HSPs in the CDSs of allohexaploid Brassica (H1) confirmed our prior findings of tolerance to temperatures as high as 40.2°C and resistance to the leaf blight pathogen *A. brassicae* (Kumari et al., 2018; Kumari et al., 2020b).

The transcriptomes are a valuable resource for developing microsatellites from the genome’s most conserved regions (Zou et al., 2013). The most promising technique to study genetic variations and marker-assisted breeding (MAB) is to employ microsatellite markers (Qi et al., 2016). Because their detection is dependent on sequencing technology, sequence completeness, mining software, and input parameters, the frequency and distribution of microsatellite markers in transcribed areas may differ between studies (Aggarwal et al., 2006; Blair and Hurtado, 2013). During the development of microsatellite markers, mononucleotides were discovered to be the most common repeat motif. In SSRs of *S. alba* transcriptomes, trinucleotides were the most prevalent repeat motif, followed by di- and mono-nucleotides, according to Zhang et al. (2016), whereas in *B. juncea* transcriptomic SSRs, dinucleotides (60.3%), and trinucleotides (38.6%) were the most abundant repeat motif types (Dhaka et al., 2017). In another investigation of microsatellite findings, trinucleotides were the most common pattern across 19 Brassicaceae species, followed by di-, tetra-, penta-, and hexa-nucleotides. Similarly, our findings for Brassicaceae and angiosperms met expectations since the most common di- and trinucleotide repeat motifs were AG/CT and AAG/CTT, respectively (Morgante et al., 2002; Victoria et al., 2011; Lopez et al., 2017). We found that the microsatellite frequency level (4.29 kb/SSR) of *S. alba* is greater than the genomic (5.88 kb/SSR) and genic (4.95 kb/SSR) SSRs previously reported (Zhang et al., 2016; Kumari et al., 2020a).

## Data Availability

The data availability statement should be replaced with following link: https://www.ncbi.nlm.nih.gov/sra/PRJNA741791; Accession No. SRR14934389.
